# Supplementary material for: Analysis of Phenolic Compounds of Reynoutria sachalinensis and Reynoutria japonica Growing in the Russian Far East
Source: Plants (Basel). 2024 Nov 27;13(23):3330. doi: 10.3390/plants13233330 (PMC11644227; doi:10.3390/plants13233330)
Supplement: Supplementary file 1 [file plants-13-03330-s001.zip › Table S5.docx]

>ITS2-1

gagagcagaaagacccgcgaacccgttcacaacacacagggggggcgcggcaccagctccgcatccctcgcgcccgggctcatctcgacaacaacacaaccccgtcgtggattggccaaggaccatgaacaatatcgcgccctgccatgcaggccttcggcgcgagcggcggtgtcgtgtcatttctacttaacaaaatgactctcaacaacggatatttcggctctcgcatcgatgaagaacgtagtgaaatacgatacttggtgtgaattgtagaattccgtgaaccatcgagtctttgaaagcatgttgcgctagaagcccttgtggccaaggcacgtctgtctgggcgtcacgcaccgcgttgcacccaccccctttggggggatcggggcagagactagccccctgtgcgctcccgcgcggccggcctaagcccatacctagtggccgtaaagcggcgcgacgattggtggtgtagcactacgcattacatcgcatcccaagtggtcccagcggccatggatgacccctcacaaaccgttgcgaccccagatc

>ITS2-2

gagagcagaaagacccgcgaacccgttcacaacacacagggggggcgcggcaccagctccgcatccctcgcgcccgggctcatctcgacaacaacacaaccccgtcgtggattggccaaggaccatgaacaatatcgcgccctgccatgcaggccttcggcgcgagcggcggtgtcgtgtcatttctacttaacaaaatgactctcaacaacggatatttcggctctcgcatcgatgaagaacgtagtgaaatacgatacttggtgtgaattgtagaattccgtgaaccatcgagtctttgaaagcatgttgcgctagaagcccttgtggccaaggcacgtctgtctgggcgtcacgcaccgcgttgcacccaccccctttggggggatcggggcagagactagccccctgtgcgctcccgcgcggccggcctaagcccatacctagtggccgtaaagcggcgcgacgattggtggtgtagcactacgcattacatcgcatcccaagtggtcccagcggccatggatgacccctcacaaaccgttgcgaccccagatc

>ITS2-3 gagagcagaaagacccgcgaacccgttcacaacacacagggggggcgcggcaccagctccgcatccctcgcgcccgggctcatctcgacaacaacacaaccccgtcgtggattggccaaggaccatgaacaatatcgcgccctgccatgcaggccttcggcgcgagcggcggtgtcgtgtcatttctacttaacaaaatgactctcaacaacggatatttcggctctcgcatcgatgaagaacgtagtgaaatacgatacttggtgtgaattgtagaattccgtgaaccatcgagtctttgaaagcatgttgcgctagaagcccttgtggccaaggcacgtctgtctgggcgtcacgcaccgcgttgcacccaccccctttggggggatcggggcagagactagccccctgtgcgctcccgcgcggccggcctaagcccatacctagtggccgtaaagcggcgcgacgattggtggtgtagcactacgcattacatcgcatcccaagtggtcccagcggccatggatgacccctcacaaaccgttgcgaccccagatc

>ITS2-4

gagagcagaaagacccgcgaacccgttcacaacacacagggggggcgcggcaccagctccgcatccctcgcgcccgggctcatctcgacaacaacacaaccccgtcgtggattggccaaggaccatgaacaatatcgcgccctgccatgcaggccttcggcgcgagcggcggtgtcgtgtcatttctacttaacaaaatgactctcaacaacggatatttcggctctcgcatcgatgaagaacgtagtgaaatacgatacttggtgtgaattgtagaattccgtgaaccatcgagtctttgaaagcatgttgcgctagaagcccttgtggccaaggcacgtctgtctgggcgtcacgcaccgcgttgcacccaccccctttggggggatcggggcagagactagccccctgtgcgctcccgcgcggccggcctaagcccatacctagtggccgtaaagcggcgcgacgattggtggtgtagcactacgcattacatcgcatcccaagtggtcccagcggccatggatgacccctcacaaaccgttgcgaccccagatc

>ITS2-5

gagagcagaaagacccgcgaacccgttcacaacacaccggggggcgcggcgccggcctcgcgcctgctccgcggcccccacgcccgggcccgtcccggcaccaacacaaccccggcgcggattgcgccaaggaccatgaacaatagcgcgccccgtcccgccggcctccggcgcgggcggcagcgtcgtgtcgtttctacttaacaaaacgactctcggcaacggatatctcggctctcgcatcgatgaagaacgtagcgaaatgcgatacttggtgtgaattgcagaatcccgtgaaccatcgagtctttgaacgcaagttgcgcccgaagccctcgtggccagggcacgtctgtctgggcgtcacgcaccgcgtcgccccctccccctccggggggtcggggcggagactggccccccgtgcgctcccgcgcgcggccggcctaaacaaagaccccgtgaccgcgaagcggcgcgacgattggtggtgtggccctgcgcatcgcgtcgcgtcccgagcggcccacggcggccacggacggccccgatcaaaccgttgcgaccccagatc

>ITS2-6

gagagcagaaagacccgcgaacccgttcacaacacaccggggggcgcggcgccggcctcgcgcctgctccgcggcccccacgcccgggcccgtcccggcaccaacacaaccccggcgcggattgcgccaaggaccatgaacaatagcgcgccccgtcccgccggcctccggcgcgggcggcagcgtcgtgtcgtttctacttaacaaaacgactctcggcaacggatatctcggctctcgcatcgatgaagaacgtagcgaaatgcgatacttggtgtgaattgcagaatcccgtgaaccatcgagtctttgaacgcaagttgcgcccgaagccctcgtggccagggcacgtctgtctgggcgtcacgcaccgcgtcgccccctccccctccggggggtcggggcggagactggccccccgtgcgctcccgcgcgcggccggcctaaacaaagaccccgtgaccgcgaagcggcgcgacgattggtggtgtggccctgcgcatcgcgtcgcgtcccgagcggcccacggcggccacggacggccccgatcaaaccgttgcgaccccagatc

>ITS2-7

gagagcagaaagacccgcgaacccgttcacaacacaccggggggcgcggcgccggcctcgcgcctgctccgcggcccccacgcccgggcccgtcccggcaccaacacaaccccggcgcggattgcgccaaggaccatgaacaatagcgcgccccgtcccgccggcctccggcgcgggcggcagcgtcgtgtcgtttctacttaacaaaacgactctcggcaacggatatctcggctctcgcatcgatgaagaacgtagcgaaatgcgatacttggtgtgaattgcagaatcccgtgaaccatcgagtctttgaacgcaagttgcgcccgaagccctcgtggccagggcacgtctgtctgggcgtcacgcaccgcgtcgccccctccccctccggggggtcggggcggagactggccccccgtgcgctcccgcgcgcggccggcctaaacaaagaccccgtgaccgcgaagcggcgcgacgattggtggtgtggccctgcgcatcgcgtcgcgtcccgagcggcccacggcggccacggacggccccgatcaaaccgttgcgaccccagatc

>ITS2-8

gagagcagaaagacccgcgaacccgttcacaacacaccggggggcgcggcgccggcctcgcgcctgctccgcggcccccacgcccgggcccgtcccggcaccaacacaaccccggcgcggattgcgccaaggaccatgaacaatagcgcgccccgtcccgccggcctccggcgcgggcggcagcgtcgtgtcgtttctacttaacaaaacgactctcggcaacggatatctcggctctcgcatcgatgaagaacgtagcgaaatgcgatacttggtgtgaattgcagaatcccgtgaaccatcgagtctttgaacgcaagttgcgcccgaagccctcgtggccagggcacgtctgtctgggcgtcacgcaccgcgtcgccccctccccctccggggggtcggggcggagactggccccccgtgcgctcccgcgcgcggccggcctaaacaaagaccccgtgaccgcgaagcggcgcgacgattggtggtgtggccctgcgcatcgcgtcgcgtcccgagcggcccacggcggccacggacggccccgatcaaaccgttgcgaccccagatc

>ITS2-9

gagagcagaaagacccgcgaacccgttcacaacacaccggggggcgcggcgccggcctcgcgcctgctccgcggcccccacgcccgggcccgtcccggcaccaacacaaccccggcgcggattgcgccaaggaccatgaacaatagcgcgccccgtcccgccggcctccggcgcgggcggcagcgtcgtgtcgtttctacttaacaaaacgactctcggcaacggatatctcggctctcgcatcgatgaagaacgtagcgaaatgcgatacttggtgtgaattgcagaatcccgtgaaccatcgagtctttgaacgcaagttgcgcccgaagccctcgtggccagggcacgtctgtctgggcgtcacgcaccgcgtcgccccctccccctccggggggtcggggcggagactggccccccgtgcgctcccgcgcgcggccggcctaaacaaagaccccgtgaccgcgaagcggcgcgacgattggtggtgtggccctgcgcatcgcgtcgcgtcccgagcggcccacggcggccacggacggccccgatcaaaccgttgcgaccccagatc

>ITS2-10

gagagcagaaagacccgcgaacccgttcacaacacaccggggggcgcggcgccggcctcgcgcctgctccgcggcccccacgcccgggcccgtcccggcaccaacacaaccccggcgcggattgcgccaaggaccatgaacaatagcgcgccccgtcccgccggcctccggcgcgggcggcagcgtcgtgtcgtttctacttaacaaaacgactctcggcaacggatatctcggctctcgcatcgatgaagaacgtagcgaaatgcgatacttggtgtgaattgcagaatcccgtgaaccatcgagtctttgaacgcaagttgcgcccgaagccctcgtggccagggcacgtctgtctgggcgtcacgcaccgcgtcgccccctccccctccggggggtcggggcggagactggccccccgtgcgctcccgcgcgcggccggcctaaacaaagaccccgtgaccgcgaagcggcgcgacgattggtggtgtggccctgcgcatcgcgtcgcgtcccgagcggcccacggcggccacggacggccccgatcaaaccgttgcgaccccagatc

>ITS2-11

gagagcagaaagacccgcgaacccgttcacaacacaccggggggcgcggcgccggcctcgcgcctgctccgcggcccccacgcccgggcccgtcccggcaccaacacaaccccggcgcggattgcgccaaggaccatgaacaatagcgcgccccgtcccgccggcctccggcgcgggcggcagcgtcgtgtcgtttctacttaacaaaacgactctcggcaacggatatctcggctctcgcatcgatgaagaacgtagcgaaatgcgatacttggtgtgaattgcagaatcccgtgaaccatcgagtctttgaacgcaagttgcgcccgaagccctcgtggccagggcacgtctgtctgggcgtcacgcaccgcgtcgccccctccccctccggggggtcggggcggagactggccccccgtgcgctcccgcgcgcggccggcctaaacaaagaccccgtgaccgcgaagcggcgcgacgattggtggtgtggccctgcgcatcgcgtcgcgtcccgagcggcccacggcggccacggacggccccgatcaaaccgttgcgaccccagatc

>ITS2-12

Gagagcagaaagacccgcgaacccgttcacaacacaccggggggcgcggcgccggcctcgcgcctgctccgcggcccccacgcccgggcccgtcccggcaccaacacaaccccggcgcggattgcgccaaggaccatgaacaatagcgcgccccgtcccgccggcctccggcgcgggcggcagcgtcgtgtcgtttctacttaacaaaacgactctcggcaacggatatctcggctctcgcatcgatgaagaacgtagcgaaatgcgatacttggtgtgaattgcagaatcccgtgaaccatcgagtctttgaacgcaagttgcgcccgaagccctcgtggccagggcacgtctgtctgggcgtcacgcaccgcgtcgccccctccccctccggggggtcggggcggagactggccccccgtgcgctcccgcgcgcggccggcctaaacaaagaccccgtgaccgcgaagcggcgcgacgattggtggtgtggccctgcgcatcgcgtcgcgtcccgagcggcccacggcggccacggacggccccgatcaaaccgttgcgaccccagatc

> ITS2-13

gagagcagaaagacccgcgaacccgttcacaacacaccggggggcgcggcgccggcctcgcgcctgctccgcggcccccacgcccgggcccgtcccggcaccaacacaaccccggcgcggattgcgccaaggaccatgaacaatagcgcgccccgtcccgccggcctccggcgcgggcggcagcgtcgtgtcgtttctacttaacaaaacgactctcggcaacggatatctcggctctcgcatcgatgaagaacgtagcgaaatgcgatacttggtgtgaattgcagaatcccgtgaaccatcgagtctttgaacgcaagttgcgcccgaagccctcgtggccagggcacgtctgtctgggcgtcacgcaccgcgtcgccccctccccctccggggggtcggggcggagactggccccccgtgcgctcccgcgcgcggccggcctaaacaaagaccccgtgaccgcgaagcggcgcgacgattggtggtgtggccctgcgcatcgcgtcgcgtcccgagcggcccacggcggccacggacggccccgatcaaaccgttgcgaccccagatc

>ITS2-14

Gagagcagaaagacccgcgaacccgttcacaacacaccggggggcgcggcgccggcctcgcgcctgctccgcggcccccacgcccgggcccgtcccggcaccaacacaaccccggcgcggattgcgccaaggaccatgaacaatagcgcgccccgtcccgccggcctccggcgcgggcggcagcgtcgtgtcgtttctacttaacaaaacgactctcggcaacggatatctcggctctcgcatcgatgaagaacgtagcgaaatgcgatacttggtgtgaattgcagaatcccgtgaaccatcgagtctttgaacgcaagttgcgcccgaagccctcgtggccagggcacgtctgtctgggcgtcacgcaccgcgtcgccccctccccctccggggggtcggggcggagactggccccccgtgcgctcccgcgcgcggccggcctaaacaaagaccccgtgaccgcgaagcggcgcgacgattggtggtgtggccctgcgcatcgcgtcgcgtcccgagcggcccacggcggccacggacggccccgatcaaaccgttgcgaccccagatc

>matK-1

ctacagtttactagttgtaaaacgtttagttattcgaatgtatcaacagaattatttgattctttctgttaatgattctaacaaaaatgaatttcttgtgcccaagaaaaatttttattctcaacagatctcagaggggtttgcagctattgcacaaattccgttttctatgcgattcatatcttccctagaaggaaaagaactaaaaaaatctcaaaatttacgaccaattcattcaacatttccttttttagaggacaaatttttacgtttaagttttgtgttagatatattgatacctcaccctgtccatctggaaatcttggttcaaactattcggtactgggtaaaagatacttcctgtttgcatttattacgattctttctttatgagtattgtaatagcgttattactctaaagagatctgtttccaatttttcaaaaaaaaagaatcaaagatttttattgttcctatataattcctatgtgtgtgaatgcgaatccatcttcgtttttctccgcaaccaatcttctcatttacgatcaacgtcttacggagcctttcttgcacgagtttatttctacctaaagttagaacattttttaaaagtatttactaagcacttcggggttatcctttggttcttcaaggatccttttctgcattctgttaggtatcaaggaaaatggattctggcttcaagagggacatttcttctgatgactaaatttaaatattactttgtcaatttctggcaatataatttttccctatgggtgcaaacaagaagactatatatcaatcaatcctcaaaccagcccacggattttatgggttttcttttaagtgtgcgactaaacccatccgtgttacggagtcaaatgttagaaaattcattcttaatagatagtggtattaagaagtttgag

>matK-2

ctacagtttactagttgtaaaacgtttagttattcgaatgtatcaacagaattatttgattctttctgttaatgattctaacaaaaatgaatttcttgtgcccaagaaaaatttttattctcaacagatctcagaggggtttgcagctattgcacaaattccgttttctatgcgattcatatcttccctagaaggaaaagaactaaaaaaatctcaaaatttacgaccaattcattcaacatttccttttttagaggacaaatttttacgtttaagttttgtgttagatatattgatacctcaccctgtccatctggaaatcttggttcaaactattcggtactgggtaaaagatacttcctgtttgcatttattacgattctttctttatgagtattgtaatagcgttattactctaaagagatctgtttccaatttttcaaaaaaaaagaatcaaagatttttattgttcctatataattcctatgtgtgtgaatgcgaatccatcttcgtttttctccgcaaccaatcttctcatttacgatcaacgtcttacggagcctttcttgcacgagtttatttctacctaaagttagaacattttttaaaagtatttactaagcacttcggggttatcctttggttcttcaaggatccttttctgcattctgttaggtatcaaggaaaatggattctggcttcaagagggacatttcttctgatgactaaatttaaatattactttgtcaatttctggcaatataatttttccctatgggtgcaaacaagaagactatatatcaatcaatcctcaaaccagcccacggattttatgggttttcttttaagtgtgcgactaaacccatccgtgttacggagtcaaatgttagaaaattcattcttaatagatagtggtattaagaagtttgag

>matK-3

ctacagtttactagttgtaaaacgtttagttattcgaatgtatcaacagaattatttgattctttctgttaatgattctaacaaaaatgaatttcttgtgcccaagaaaaatttttattctcaacagatctcagaggggtttgcagctattgcacaaattccgttttctatgcgattcatatcttccctagaaggaaaagaactaaaaaaatctcaaaatttacgaccaattcattcaacatttccttttttagaggacaaatttttacgtttaagttttgtgttagatatattgatacctcaccctgtccatctggaaatcttggttcaaactattcggtactgggtaaaagatacttcctgtttgcatttattacgattctttctttatgagtattgtaatagcgttattactctaaagagatctgtttccaatttttcaaaaaaaaagaatcaaagatttttattgttcctatataattcctatgtgtgtgaatgcgaatccatcttcgtttttctccgcaaccaatcttctcatttacgatcaacgtcttacggagcctttcttgcacgagtttatttctacctaaagttagaacattttttaaaagtatttactaagcacttcggggttatcctttggttcttcaaggatccttttctgcattctgttaggtatcaaggaaaatggattctggcttcaagagggacatttcttctgatgactaaatttaaatattactttgtcaatttctggcaatataatttttccctatgggtgcaaacaagaagactatatatcaatcaatcctcaaaccagcccacggattttatgggttttcttttaagtgtgcgactaaacccatccgtgttacggagtcaaatgttagaaaattcattcttaatagatagtggtattaagaagtttgag

>matK-4

ctacagtttactagttgtaaaacgtttagttattcgaatgtatcaacagaattatttgattctttctgttaatgattctaacaaaaatgaatttcttgtgcccaagaaaaatttttattctcaacagatctcagaggggtttgcagctattgcacaaattccgttttctatgcgattcatatcttccctagaaggaaaagaactaaaaaaatctcaaaatttacgaccaattcattcaacatttccttttttagaggacaaatttttacgtttaagttttgtgttagatatattgatacctcaccctgtccatctggaaatcttggttcaaactattcggtactgggtaaaagatacttcctgtttgcatttattacgattctttctttatgagtattgtaatagcgttattactctaaagagatctgtttccaatttttcaaaaaaaaagaatcaaagatttttattgttcctatataattcctatgtgtgtgaatgcgaatccatcttcgtttttctccgcaaccaatcttctcatttacgatcaacgtcttacggagcctttcttgcacgagtttatttctacctaaagttagaacattttttaaaagtatttactaagcacttcggggttatcctttggttcttcaaggatccttttctgcattctgttaggtatcaaggaaaatggattctggcttcaagagggacatttcttctgatgactaaatttaaatattactttgtcaatttctggcaatataatttttccctatgggtgcaaacaagaagactatatatcaatcaatcctcaaaccagcccacggattttatgggttttcttttaagtgtgcgactaaacccatccgtgttacggagtcaaatgttagaaaattcattcttaatagatagtggtattaagaagtttgag

>matK-5

ctacagtttactagttgtaaaacgtttagttattcgaatgtatcaacagaattatttgattctttctgttaatgattctaacaaaaatgaatttcttgggcacaagaaaaatttttattctcaacagatctcagaggggtttgcagctattgcgcaaattccgttttctatgcgattaatatcttccctagaaggaaaagaactaaaaaaatctcaaaatttacgaccaattcattcaacatttccttttttagaggacaaatttttacgtttaagttttgtgttagatatattgatacctcaccctgtccatctggaaatcttggttcaaactattcggtactgggtaaaagatacctcctgtttgcatttattacgattctttctttatgagtattgtaatagcgttattactctaaagagatctgtttccaatttttcaaaaaaaaagaatcaaagatttttattgttcctatataattcctatgtgtgtgaatgcgaatccatcttcgtttttctccgcaaccaaccttctcatttacgatcaacgtcttacggagcctttcttgcacgagtttatttctacctaaagttagaacattttttaaaagctaagcacttcggggttatcctttggttcttcaaggatccttttctgcattctgttaggtatcaaggaaaatggattctggcttcaagagggacatttcttctgatgactaaatttaaatattactttgtcaatttctggcaatataatttttccctatgggtgcaaacaagaagactctatatcaatcaaccctcaaaccagcccacgaattttatgggttttcttttaagtgtgcgactaaacccatctgtgttacggagtcaaatgttagaaaattcattcttaatagatagtggtattaagaagtttgag

>matK-6

ctacagtttactagttgtaaaacgtttagttattcgaatgtatcaacagaattatttgattctttctgttaatgattctaacaaaaatgaatttcttgggcacaagaaaaatttttattctcaacagatctcagaggggtttgcagctattgcgcaaattccgttttctatgcgattaatatcttccctagaaggaaaagaactaaaaaaatctcaaaatttacgaccaattcattcaacatttccttttttagaggacaaatttttacgtttaagttttgtgttagatatattgatacctcaccctgtccatctggaaatcttggttcaaactattcggtactgggtaaaagatacctcctgtttgcatttattacgattctttctttatgagtattgtaatagcgttattactctaaagagatctgtttccaatttttcaaaaaaaaagaatcaaagatttttattgttcctatataattcctatgtgtgtgaatgcgaatccatcttcgtttttctccgcaaccaaccttctcatttacgatcaacgtcttacggagcctttcttgcacgagtttatttctacctaaagttagaacattttttaaaagctaagcacttcggggttatcctttggttcttcaaggatccttttctgcattctgttaggtatcaaggaaaatggattctggcttcaagagggacatttcttctgatgactaaatttaaatattactttgtcaatttctggcaatataatttttccctatgggtgcaaacaagaagactctatatcaatcaaccctcaaaccagcccacgaattttatgggttttcttttaagtgtgcgactaaacccatctgtgttacggagtcaaatgttagaaaattcattcttaatagatagtggtattaagaagtttgag

>matK-7

ctacagtttactagttgtaaaacgtttagttattcgaatgtatcaacagaattatttgattctttctgttaatgattctaacaaaaatgaatttcttgggcacaagaaaaatttttattctcaacagatctcagaggggtttgcagctattgcgcaaattccgttttctatgcgattaatatcttccctagaaggaaaagaactaaaaaaatctcaaaatttacgaccaattcattcaacatttccttttttagaggacaaatttttacgtttaagttttgtgttagatatattgatacctcaccctgtccatctggaaatcttggttcaaactattcggtactgggtaaaagatacctcctgtttgcatttattacgattctttctttatgagtattgtaatagcgttattactctaaagagatctgtttccaatttttcaaaaaaaaagaatcaaagatttttattgttcctatataattcctatgtgtgtgaatgcgaatccatcttcgtttttctccgcaaccaaccttctcatttacgatcaacgtcttacggagcctttcttgcacgagtttatttctacctaaagttagaacattttttaaaagctaagcacttcggggttatcctttggttcttcaaggatccttttctgcattctgttaggtatcaaggaaaatggattctggcttcaagagggacatttcttctgatgactaaatttaaatattactttgtcaatttctggcaatataatttttccctatgggtgcaaacaagaagactctatatcaatcaaccctcaaaccagcccacgaattttatgggttttcttttaagtgtgcgactaaacccatctgtgttacggagtcaaatgttagaaaattcattcttaatagatagtggtattaagaagtttgag

>matK-8

ctacagtttactagttgtaaaacgtttagttattcgaatgtatcaacagaattatttgattctttctgttaatgattctaacaaaaatgaatttcttgggcacaagaaaaatttttattctcaacagatctcagaggggtttgcagctattgcgcaaattccgttttctatgcgattaatatcttccctagaaggaaaagaactaaaaaaatctcaaaatttacgaccaattcattcaacatttccttttttagaggacaaatttttacgtttaagttttgtgttagatatattgatacctcaccctgtccatctggaaatcttggttcaaactattcggtactgggtaaaagatacctcctgtttgcatttattacgattctttctttatgagtattgtaatagcgttattactctaaagagatctgtttccaatttttcaaaaaaaaagaatcaaagatttttattgttcctatataattcctatgtgtgtgaatgcgaatccatcttcgtttttctccgcaaccaaccttctcatttacgatcaacgtcttacggagcctttcttgcacgagtttatttctacctaaagttagaacattttttaaaagctaagcacttcggggttatcctttggttcttcaaggatccttttctgcattctgttaggtatcaaggaaaatggattctggcttcaagagggacatttcttctgatgactaaatttaaatattactttgtcaatttctggcaatataatttttccctatgggtgcaaacaagaagactctatatcaatcaaccctcaaaccagcccacgaattttatgggttttcttttaagtgtgcgactaaacccatctgtgttacggagtcaaatgttagaaaattcattcttaatagatagtggtattaagaagtttgag

>matK-9

ctacagtttactagttgtaaaacgtttagttattcgaatgtatcaacagaattatttgattctttctgttaatgattctaacaaaaatgaatttcttgggcacaagaaaaatttttattctcaacagatctcagaggggtttgcagctattgcgcaaattccgttttctatgcgattaatatcttccctagaaggaaaagaactaaaaaaatctcaaaatttacgaccaattcattcaacatttccttttttagaggacaaatttttacgtttaagttttgtgttagatatattgatacctcaccctgtccatctggaaatcttggttcaaactattcggtactgggtaaaagatacctcctgtttgcatttattacgattctttctttatgagtattgtaatagcgttattactctaaagagatctgtttccaatttttcaaaaaaaaagaatcaaagatttttattgttcctatataattcctatgtgtgtgaatgcgaatccatcttcgtttttctccgcaaccaaccttctcatttacgatcaacgtcttacggagcctttcttgcacgagtttatttctacctaaagttagaacattttttaaaagctaagcacttcggggttatcctttggttcttcaaggatccttttctgcattctgttaggtatcaaggaaaatggattctggcttcaagagggacatttcttctgatgactaaatttaaatattactttgtcaatttctggcaatataatttttccctatgggtgcaaacaagaagactctatatcaatcaaccctcaaaccagcccacgaattttatgggttttcttttaagtgtgcgactaaacccatctgtgttacggagtcaaatgttagaaaattcattcttaatagatagtggtattaagaagtttgag

>matK-10

ctacagtttactagttgtaaaacgtttagttattcgaatgtatcaacagaattatttgattctttctgttaatgattctaacaaaaatgaatttcttgggcacaagaaaaatttttattctcaacagatctcagaggggtttgcagctattgcgcaaattccgttttctatgcgattaatatcttccctagaaggaaaagaactaaaaaaatctcaaaatttacgaccaattcattcaacatttccttttttagaggacaaatttttacgtttaagttttgtgttagatatattgatacctcaccctgtccatctggaaatcttggttcaaactattcggtactgggtaaaagatacctcctgtttgcatttattacgattctttctttatgagtattgtaatagcgttattactctaaagagatctgtttccaatttttcaaaaaaaaagaatcaaagatttttattgttcctatataattcctatgtgtgtgaatgcgaatccatcttcgtttttctccgcaaccaaccttctcatttacgatcaacgtcttacggagcctttcttgcacgagtttatttctacctaaagttagaacattttttaaaagctaagcacttcggggttatcctttggttcttcaaggatccttttctgcattctgttaggtatcaaggaaaatggattctggcttcaagagggacatttcttctgatgactaaatttaaatattactttgtcaatttctggcaatataatttttccctatgggtgcaaacaagaagactctatatcaatcaaccctcaaaccagcccacgaattttatgggttttcttttaagtgtgcgactaaacccatctgtgttacggagtcaaatgttagaaaattcattcttaatagatagtggtattaagaagtttgag

>matK-11

ctacagtttactagttgtaaaacgtttagttattcgaatgtatcaacagaattatttgattctttctgttaatgattctaacaaaaatgaatttcttgggcacaagaaaaatttttattctcaacagatctcagaggggtttgcagctattgcgcaaattccgttttctatgcgattaatatcttccctagaaggaaaagaactaaaaaaatctcaaaatttacgaccaattcattcaacatttccttttttagaggacaaatttttacgtttaagttttgtgttagatatattgatacctcaccctgtccatctggaaatcttggttcaaactattcggtactgggtaaaagatacctcctgtttgcatttattacgattctttctttatgagtattgtaatagcgttattactctaaagagatctgtttccaatttttcaaaaaaaaagaatcaaagatttttattgttcctatataattcctatgtgtgtgaatgcgaatccatcttcgtttttctccgcaaccaaccttctcatttacgatcaacgtcttacggagcctttcttgcacgagtttatttctacctaaagttagaacattttttaaaagctaagcacttcggggttatcctttggttcttcaaggatccttttctgcattctgttaggtatcaaggaaaatggattctggcttcaagagggacatttcttctgatgactaaatttaaatattactttgtcaatttctggcaatataatttttccctatgggtgcaaacaagaagactctatatcaatcaaccctcaaaccagcccacgaattttatgggttttcttttaagtgtgcgactaaacccatctgtgttacggagtcaaatgttagaaaattcattcttaatagatagtggtattaagaagtttgag

>matK-12

ctacagtttactagttgtaaaacgtttagttattcgaatgtatcaacagaattatttgattctttctgttaatgattctaacaaaaatgaatttcttgggcacaagaaaaatttttattctcaacagatctcagaggggtttgcagctattgcgcaaattccgttttctatgcgattaatatcttccctagaaggaaaagaactaaaaaaatctcaaaatttacgaccaattcattcaacatttccttttttagaggacaaatttttacgtttaagttttgtgttagatatattgatacctcaccctgtccatctggaaatcttggttcaaactattcggtactgggtaaaagatacctcctgtttgcatttattacgattctttctttatgagtattgtaatagcgttattactctaaagagatctgtttccaatttttcaaaaaaaaagaatcaaagatttttattgttcctatataattcctatgtgtgtgaatgcgaatccatcttcgtttttctccgcaaccaaccttctcatttacgatcaacgtcttacggagcctttcttgcacgagtttatttctacctaaagttagaacattttttaaaagctaagcacttcggggttatcctttggttcttcaaggatccttttctgcattctgttaggtatcaaggaaaatggattctggcttcaagagggacatttcttctgatgactaaatttaaatattactttgtcaatttctggcaatataatttttccctatgggtgcaaacaagaagactctatatcaatcaaccctcaaaccagcccacgaattttatgggttttcttttaagtgtgcgactaaacccatctgtgttacggagtcaaatgttagaaaattcattcttaatagatagtggtattaagaagtttgag

>matK-13

ctacagtttactagttgtaaaacgtttagttattcgaatgtatcaacagaattatttgattctttctgttaatgattctaacaaaaatgaatttcttgggcacaagaaaaatttttattctcaacagatctcagaggggtttgcagctattgcgcaaattccgttttctatgcgattaatatcttccctagaaggaaaagaactaaaaaaatctcaaaatttacgaccaattcattcaacatttccttttttagaggacaaatttttacgtttaagttttgtgttagatatattgatacctcaccctgtccatctggaaatcttggttcaaactattcggtactgggtaaaagatacctcctgtttgcatttattacgattctttctttatgagtattgtaatagcgttattactctaaagagatctgtttccaatttttcaaaaaaaaagaatcaaagatttttattgttcctatataattcctatgtgtgtgaatgcgaatccatcttcgtttttctccgcaaccaaccttctcatttacgatcaacgtcttacggagcctttcttgcacgagtttatttctacctaaagttagaacattttttaaaagctaagcacttcggggttatcctttggttcttcaaggatccttttctgcattctgttaggtatcaaggaaaatggattctggcttcaagagggacatttcttctgatgactaaatttaaatattactttgtcaatttctggcaatataatttttccctatgggtgcaaacaagaagactctatatcaatcaaccctcaaaccagcccacgaattttatgggttttcttttaagtgtgcgactaaacccatctgtgttacggagtcaaatgttagaaaattcattcttaatagatagtggtattaagaagtttgag

>matK-14

Ctacagtttactagttgtaaaacgtttagttattcgaatgtatcaacagaattatttgattctttctgttaatgattctaacaaaaatgaatttcttgggcacaagaaaaatttttattctcaacagatctcagaggggtttgcagctattgcgcaaattccgttttctatgcgattaatatcttccctagaaggaaaagaactaaaaaaatctcaaaatttacgaccaattcattcaacatttccttttttagaggacaaatttttacgtttaagttttgtgttagatatattgatacctcaccctgtccatctggaaatcttggttcaaactattcggtactgggtaaaagatacctcctgtttgcatttattacgattctttctttatgagtattgtaatagcgttattactctaaagagatctgtttccaatttttcaaaaaaaaagaatcaaagatttttattgttcctatataattcctatgtgtgtgaatgcgaatccatcttcgtttttctccgcaaccaaccttctcatttacgatcaacgtcttacggagcctttcttgcacgagtttatttctacctaaagttagaacattttttaaaagctaagcacttcggggttatcctttggttcttcaaggatccttttctgcattctgttaggtatcaaggaaaatggattctggcttcaagagggacatttcttctgatgactaaatttaaatattactttgtcaatttctggcaatataatttttccctatgggtgcaaacaagaagactctatatcaatcaaccctcaaaccagcccacgaattttatgggttttcttttaagtgtgcgactaaacccatctgtgttacggagtcaaatgttagaaaattcattcttaatagatagtggtattaagaagtttgag

>rps16-1

TGATCAAAATAAAAATTTTTGATATAAGATGTCTAGAAAAAAGGATCGAGTAAATAAACCAAGCAAGTTTTATATTGACCAAATAACTAACGGTAGGAATTGGAGCTAGCAATAAACAATTCTGACATTTATTTCAATTGGATGGTTTTCTTTGGAACTATACGCAAAAAGAATTCATTATTTTATTTGACGTATAATTTTTGATATTTTTTCTACTTCTTTTGTATTTCGCTCTACATTCCTTTCTGATCATCTACCTTATTTAGCTAATTTAGATAGTGCCAATCCAACACAAGTTCTTTTTTTTTAACATACATATTGACAAGAGTGTAGTGAACAAATATAATTCAAGTGTAAATGTATCCGTTTTTCTCTATTTTTTTGTCCTACATACAAAACACAGGTTTTGTTTTTTACTTTATTCAAAGATTTGTTGAATTTGTTATGATACAAAACAAAACTGCTTATAAGGAAATGGATAGATAATAAAAACAAAACTGCTTATAAGGAAATGGATAGATAATCAAAAAAAATATCTGAAAATATAGAGCTAGAAAGATAGAGAAAAAAAATAGAATAAGAATATCTATCTATAATATAATGTAGTGGATGAAAATATCTAAGAAACAGTCTAGTTTTTTATTTGAAAATTTCTTGTATTGTCAGTTGATCTTTTTTTTTCTTTT

>rps16-2

TGATCAAAATAAAAATTTTTGATATAAGATGTCTAGAAAAAAGGATCGAGTAAATAAACCAAGCAAGTTTTATATTGACCAAATAACTAACGGTAGGAATTGGAGCTAGCAATAAACAATTCTGACATTTATTTCAATTGGATGGTTTTCTTTGGAACTATACGCAAAAAGAATTCATTATTTTATTTGACGTATAATTTTTGATATTTTTTCTACTTCTTTTGTATTTCGCTCTACATTCCTTTCTGATCATCTACCTTATTTAGCTAATTTAGATAGTGCCAATCCAACACAAGTTCTTTTTTTTTAACATACATATTGACAAGAGTGTAGTGAACAAATATAATTCAAGTGTAAATGTATCCGTTTTTCTCTATTTTTTTGTCCTACATACAAAACACAGGTTTTGTTTTTTACTTTATTCAAAGATTTGTTGAATTTGTTATGATACAAAACAAAACTGCTTATAAGGAAATGGATAGATAATAAAAACAAAACTGCTTATAAGGAAATGGATAGATAATCAAAAAAAATATCTGAAAATATAGAGCTAGAAAGATAGAGAAAAAAAATAGAATAAGAATATCTATCTATAATATAATGTAGTGGATGAAAATATCTAAGAAACAGTCTAGTTTTTTATTTGAAAATTTCTTGTATTGTCAGTTGATCTTTTTTTTTCTTTT

>rps16-3

TGATCAAAATAAAAATTTTTGATATAAGATGTCTAGAAAAAAGGATCGAGTAAATAAACCAAGCAAGTTTTATATTGACCAAATAACTAACGGTAGGAATTGGAGCTAGCAATAAACAATTCTGACATTTATTTCAATTGGATGGTTTTCTTTGGAACTATACGCAAAAAGAATTCATTATTTTATTTGACGTATAATTTTTGATATTTTTTCTACTTCTTTTGTATTTCGCTCTACATTCCTTTCTGATCATCTACCTTATTTAGCTAATTTAGATAGTGCCAATCCAACACAAGTTCTTTTTTTTTAACATACATATTGACAAGAGTGTAGTGAACAAATATAATTCAAGTGTAAATGTATCCGTTTTTCTCTATTTTTTTGTCCTACATACAAAACACAGGTTTTGTTTTTTACTTTATTCAAAGATTTGTTGAATTTGTTATGATACAAAACAAAACTGCTTATAAGGAAATGGATAGATAATAAAAACAAAACTGCTTATAAGGAAATGGATAGATAATCAAAAAAAATATCTGAAAATATAGAGCTAGAAAGATAGAGAAAAAAAATAGAATAAGAATATCTATCTATAATATAATGTAGTGGATGAAAATATCTAAGAAACAGTCTAGTTTTTTATTTGAAAATTTCTTGTATTGTCAGTTGATCTTTTTTTTTCTTTT

>rps16-4

TGATCAAAATAAAAATTTTTGATATAAGATGTCTAGAAAAAAGGATCGAGTAAATAAACCAAGCAAGTTTTATATTGACCAAATAACTAACGGTAGGAATTGGAGCTAGCAATAAACAATTCTGACATTTATTTCAATTGGATGGTTTTCTTTGGAACTATACGCAAAAAGAATTCATTATTTTATTTGACGTATAATTTTTGATATTTTTTCTACTTCTTTTGTATTTCGCTCTACATTCCTTTCTGATCATCTACCTTATTTAGCTAATTTAGATAGTGCCAATCCAACACAAGTTCTTTTTTTTTAACATACATATTGACAAGAGTGTAGTGAACAAATATAATTCAAGTGTAAATGTATCCGTTTTTCTCTATTTTTTTGTCCTACATACAAAACACAGGTTTTGTTTTTTACTTTATTCAAAGATTTGTTGAATTTGTTATGATACAAAACAAAACTGCTTATAAGGAAATGGATAGATAATAAAAACAAAACTGCTTATAAGGAAATGGATAGATAATCAAAAAAAATATCTGAAAATATAGAGCTAGAAAGATAGAGAAAAAAAATAGAATAAGAATATCTATCTATAATATAATGTAGTGGATGAAAATATCTAAGAAACAGTCTAGTTTTTTATTTGAAAATTTCTTGTATTGTCAGTTGATCTTTTTTTTTCTTTT

>rps16-5

AATTTTTGATATAAGATGTCTAGAAAAAAGGATCAAGTAAATAAACCAAGCAAGTTTTATATTGACCAAATAACTAACGGTAGGAATTGGAGCTAGCAATAAACAATTCTGACATTTATTTCAATTGGATGGTTTTCTTTGGAACTATACGCAAAAAGAATTCATTATTTTATTTGACGTATAATTTTTGATATTTTTTCTACTTCTTTTGTATTTCGCTCTACATTCCTTTCTGATCATCTACCTTATTTAGCTAATTTAGATAGTGCCAATCCAACACAAGTTCTTTTTTTTTTATTTGTTCAATTTCATTGATTATTTTCGTATCTTAAATTTTCAATGAAATTTTTATAATTTCGATTTTTCGTTCTATATATTTATTCTTTTTTTTTTAACATACATATTGACAAGAGTGTAGTGAACAAATATAATTCAAGTGTAAATGGATCCGTTTTTCTCTATTTTTTTGTCCTACATACAAAACACAGGTTTTGTTTTTTACTTTATTCAAAGATTTGTTGAATTTGTTATGATACAAAACAAAACTGCTTATAAGGAAATGGATAGATAATAAAAAAAAAATATCTGAAAATATAGAGATAGAAAGATAGAGAAAAAAAATAGAATAAGAATATATATCTATCTATAATGTAGTGGATGAAAATATCTAAGAAACAGTCTAGTTTTTTATTTGAAAATTTCTTGTATTGTCAGTTGATCTTTTTTTTTCTTTTTTGCT

>rps16-6

AATTTTTGATATAAGATGTCTAGAAAAAAGGATCAAGTAAATAAACCAAGCAAGTTTTATATTGACCAAATAACTAACGGTAGGAATTGGAGCTAGCAATAAACAATTCTGACATTTATTTCAATTGGATGGTTTTCTTTGGAACTATACGCAAAAAGAATTCATTATTTTATTTGACGTATAATTTTTGATATTTTTTCTACTTCTTTTGTATTTCGCTCTACATTCCTTTCTGATCATCTACCTTATTTAGCTAATTTAGATAGTGCCAATCCAACACAAGTTCTTTTTTTTTTATTTGTTCAATTTCATTGATTATTTTCGTATCTTAAATTTTCAATGAAATTTTTATAATTTCGATTTTTCGTTCTATATATTTATTCTTTTTTTTTTAACATACATATTGACAAGAGTGTAGTGAACAAATATAATTCAAGTGTAAATGGATCCGTTTTTCTCTATTTTTTTGTCCTACATACAAAACACAGGTTTTGTTTTTTACTTTATTCAAAGATTTGTTGAATTTGTTATGATACAAAACAAAACTGCTTATAAGGAAATGGATAGATAATAAAAAAAAAATATCTGAAAATATAGAGATAGAAAGATAGAGAAAAAAAATAGAATAAGAATATATATCTATCTATAATGTAGTGGATGAAAATATCTAAGAAACAGTCTAGTTTTTTATTTGAAAATTTCTTGTATTGTCAGTTGATCTTTTTTTTTCTTTTTTGCT

>rps16-7

AATTTTTGATATAAGATGTCTAGAAAAAAGGATCAAGTAAATAAACCAAGCAAGTTTTATATTGACCAAATAACTAACGGTAGGAATTGGAGCTAGCAATAAACAATTCTGACATTTATTTCAATTGGATGGTTTTCTTTGGAACTATACGCAAAAAGAATTCATTATTTTATTTGACGTATAATTTTTGATATTTTTTCTACTTCTTTTGTATTTCGCTCTACATTCCTTTCTGATCATCTACCTTATTTAGCTAATTTAGATAGTGCCAATCCAACACAAGTTCTTTTTTTTTTATTTGTTCAATTTCATTGATTATTTTCGTATCTTAAATTTTCAATGAAATTTTTATAATTTCGATTTTTCGTTCTATATATTTATTCTTTTTTTTTTAACATACATATTGACAAGAGTGTAGTGAACAAATATAATTCAAGTGTAAATGGATCCGTTTTTCTCTATTTTTTTGTCCTACATACAAAACACAGGTTTTGTTTTTTACTTTATTCAAAGATTTGTTGAATTTGTTATGATACAAAACAAAACTGCTTATAAGGAAATGGATAGATAATAAAAAAAAAATATCTGAAAATATAGAGATAGAAAGATAGAGAAAAAAAATAGAATAAGAATATATATCTATCTATAATGTAGTGGATGAAAATATCTAAGAAACAGTCTAGTTTTTTATTTGAAAATTTCTTGTATTGTCAGTTGATCTTTTTTTTTCTTTTTTGCT

>rps16-8

AATTTTTGATATAAGATGTCTAGAAAAAAGGATCAAGTAAATAAACCAAGCAAGTTTTATATTGACCAAATAACTAACGGTAGGAATTGGAGCTAGCAATAAACAATTCTGACATTTATTTCAATTGGATGGTTTTCTTTGGAACTATACGCAAAAAGAATTCATTATTTTATTTGACGTATAATTTTTGATATTTTTTCTACTTCTTTTGTATTTCGCTCTACATTCCTTTCTGATCATCTACCTTATTTAGCTAATTTAGATAGTGCCAATCCAACACAAGTTCTTTTTTTTTTATTTGTTCAATTTCATTGATTATTTTCGTATCTTAAATTTTCAATGAAATTTTTATAATTTCGATTTTTCGTTCTATATATTTATTCTTTTTTTTTTAACATACATATTGACAAGAGTGTAGTGAACAAATATAATTCAAGTGTAAATGGATCCGTTTTTCTCTATTTTTTTGTCCTACATACAAAACACAGGTTTTGTTTTTTACTTTATTCAAAGATTTGTTGAATTTGTTATGATACAAAACAAAACTGCTTATAAGGAAATGGATAGATAATAAAAAAAAAATATCTGAAAATATAGAGATAGAAAGATAGAGAAAAAAAATAGAATAAGAATATATATCTATCTATAATGTAGTGGATGAAAATATCTAAGAAACAGTCTAGTTTTTTATTTGAAAATTTCTTGTATTGTCAGTTGATCTTTTTTTTTCTTTTTTGCT

>rps16-9

AATTTTTGATATAAGATGTCTAGAAAAAAGGATCAAGTAAATAAACCAAGCAAGTTTTATATTGACCAAATAACTAACGGTAGGAATTGGAGCTAGCAATAAACAATTCTGACATTTATTTCAATTGGATGGTTTTCTTTGGAACTATACGCAAAAAGAATTCATTATTTTATTTGACGTATAATTTTTGATATTTTTTCTACTTCTTTTGTATTTCGCTCTACATTCCTTTCTGATCATCTACCTTATTTAGCTAATTTAGATAGTGCCAATCCAACACAAGTTCTTTTTTTTTTATTTGTTCAATTTCATTGATTATTTTCGTATCTTAAATTTTCAATGAAATTTTTATAATTTCGATTTTTCGTTCTATATATTTATTCTTTTTTTTTTAACATACATATTGACAAGAGTGTAGTGAACAAATATAATTCAAGTGTAAATGGATCCGTTTTTCTCTATTTTTTTGTCCTACATACAAAACACAGGTTTTGTTTTTTACTTTATTCAAAGATTTGTTGAATTTGTTATGATACAAAACAAAACTGCTTATAAGGAAATGGATAGATAATAAAAAAAAAATATCTGAAAATATAGAGATAGAAAGATAGAGAAAAAAAATAGAATAAGAATATATATCTATCTATAATGTAGTGGATGAAAATATCTAAGAAACAGTCTAGTTTTTTATTTGAAAATTTCTTGTATTGTCAGTTGATCTTTTTTTTTCTTTTTTGCT

>rps16-10

AATTTTTGATATAAGATGTCTAGAAAAAAGGATCAAGTAAATAAACCAAGCAAGTTTTATATTGACCAAATAACTAACGGTAGGAATTGGAGCTAGCAATAAACAATTCTGACATTTATTTCAATTGGATGGTTTTCTTTGGAACTATACGCAAAAAGAATTCATTATTTTATTTGACGTATAATTTTTGATATTTTTTCTACTTCTTTTGTATTTCGCTCTACATTCCTTTCTGATCATCTACCTTATTTAGCTAATTTAGATAGTGCCAATCCAACACAAGTTCTTTTTTTTTTATTTGTTCAATTTCATTGATTATTTTCGTATCTTAAATTTTCAATGAAATTTTTATAATTTCGATTTTTCGTTCTATATATTTATTCTTTTTTTTTTAACATACATATTGACAAGAGTGTAGTGAACAAATATAATTCAAGTGTAAATGGATCCGTTTTTCTCTATTTTTTTGTCCTACATACAAAACACAGGTTTTGTTTTTTACTTTATTCAAAGATTTGTTGAATTTGTTATGATACAAAACAAAACTGCTTATAAGGAAATGGATAGATAATAAAAAAAAAATATCTGAAAATATAGAGATAGAAAGATAGAGAAAAAAAATAGAATAAGAATATATATCTATCTATAATGTAGTGGATGAAAATATCTAAGAAACAGTCTAGTTTTTTATTTGAAAATTTCTTGTATTGTCAGTTGATCTTTTTTTTTCTTTTTTGCT

>rps16-11

AATTTTTGATATAAGATGTCTAGAAAAAAGGATCAAGTAAATAAACCAAGCAAGTTTTATATTGACCAAATAACTAACGGTAGGAATTGGAGCTAGCAATAAACAATTCTGACATTTATTTCAATTGGATGGTTTTCTTTGGAACTATACGCAAAAAGAATTCATTATTTTATTTGACGTATAATTTTTGATATTTTTTCTACTTCTTTTGTATTTCGCTCTACATTCCTTTCTGATCATCTACCTTATTTAGCTAATTTAGATAGTGCCAATCCAACACAAGTTCTTTTTTTTTTATTTGTTCAATTTCATTGATTATTTTCGTATCTTAAATTTTCAATGAAATTTTTATAATTTCGATTTTTCGTTCTATATATTTATTCTTTTTTTTTTAACATACATATTGACAAGAGTGTAGTGAACAAATATAATTCAAGTGTAAATGGATCCGTTTTTCTCTATTTTTTTGTCCTACATACAAAACACAGGTTTTGTTTTTTACTTTATTCAAAGATTTGTTGAATTTGTTATGATACAAAACAAAACTGCTTATAAGGAAATGGATAGATAATAAAAAAAAAATATCTGAAAATATAGAGATAGAAAGATAGAGAAAAAAAATAGAATAAGAATATATATCTATCTATAATGTAGTGGATGAAAATATCTAAGAAACAGTCTAGTTTTTTATTTGAAAATTTCTTGTATTGTCAGTTGATCTTTTTTTTTCTTTTTTGCT

>rps16-12

AATTTTTGATATAAGATGTCTAGAAAAAAGGATCAAGTAAATAAACCAAGCAAGTTTTATATTGACCAAATAACTAACGGTAGGAATTGGAGCTAGCAATAAACAATTCTGACATTTATTTCAATTGGATGGTTTTCTTTGGAACTATACGCAAAAAGAATTCATTATTTTATTTGACGTATAATTTTTGATATTTTTTCTACTTCTTTTGTATTTCGCTCTACATTCCTTTCTGATCATCTACCTTATTTAGCTAATTTAGATAGTGCCAATCCAACACAAGTTCTTTTTTTTTTATTTGTTCAATTTCATTGATTATTTTCGTATCTTAAATTTTCAATGAAATTTTTATAATTTCGATTTTTCGTTCTATATATTTATTCTTTTTTTTTTAACATACATATTGACAAGAGTGTAGTGAACAAATATAATTCAAGTGTAAATGGATCCGTTTTTCTCTATTTTTTTGTCCTACATACAAAACACAGGTTTTGTTTTTTACTTTATTCAAAGATTTGTTGAATTTGTTATGATACAAAACAAAACTGCTTATAAGGAAATGGATAGATAATAAAAAAAAAATATCTGAAAATATAGAGATAGAAAGATAGAGAAAAAAAATAGAATAAGAATATATATCTATCTATAATGTAGTGGATGAAAATATCTAAGAAACAGTCTAGTTTTTTATTTGAAAATTTCTTGTATTGTCAGTTGATCTTTTTTTTTCTTTTTTGCT

>rps16-13

AATTTTTGATATAAGATGTCTAGAAAAAAGGATCAAGTAAATAAACCAAGCAAGTTTTATATTGACCAAATAACTAACGGTAGGAATTGGAGCTAGCAATAAACAATTCTGACATTTATTTCAATTGGATGGTTTTCTTTGGAACTATACGCAAAAAGAATTCATTATTTTATTTGACGTATAATTTTTGATATTTTTTCTACTTCTTTTGTATTTCGCTCTACATTCCTTTCTGATCATCTACCTTATTTAGCTAATTTAGATAGTGCCAATCCAACACAAGTTCTTTTTTTTTTATTTGTTCAATTTCATTGATTATTTTCGTATCTTAAATTTTCAATGAAATTTTTATAATTTCGATTTTTCGTTCTATATATTTATTCTTTTTTTTTTAACATACATATTGACAAGAGTGTAGTGAACAAATATAATTCAAGTGTAAATGGATCCGTTTTTCTCTATTTTTTTGTCCTACATACAAAACACAGGTTTTGTTTTTTACTTTATTCAAAGATTTGTTGAATTTGTTATGATACAAAACAAAACTGCTTATAAGGAAATGGATAGATAATAAAAAAAAAATATCTGAAAATATAGAGATAGAAAGATAGAGAAAAAAAATAGAATAAGAATATATATCTATCTATAATGTAGTGGATGAAAATATCTAAGAAACAGTCTAGTTTTTTATTTGAAAATTTCTTGTATTGTCAGTTGATCTTTTTTTTTCTTTTTTGCT

>rps16-14

AATTTTTGATATAAGATGTCTAGAAAAAAGGATCAAGTAAATAAACCAAGCAAGTTTTATATTGACCAAATAACTAACGGTAGGAATTGGAGCTAGCAATAAACAATTCTGACATTTATTTCAATTGGATGGTTTTCTTTGGAACTATACGCAAAAAGAATTCATTATTTTATTTGACGTATAATTTTTGATATTTTTTCTACTTCTTTTGTATTTCGCTCTACATTCCTTTCTGATCATCTACCTTATTTAGCTAATTTAGATAGTGCCAATCCAACACAAGTTCTTTTTTTTTTATTTGTTCAATTTCATTGATTATTTTCGTATCTTAAATTTTCAATGAAATTTTTATAATTTCGATTTTTCGTTCTATATATTTATTCTTTTTTTTTTAACATACATATTGACAAGAGTGTAGTGAACAAATATAATTCAAGTGTAAATGGATCCGTTTTTCTCTATTTTTTTGTCCTACATACAAAACACAGGTTTTGTTTTTTACTTTATTCAAAGATTTGTTGAATTTGTTATGATACAAAACAAAACTGCTTATAAGGAAATGGATAGATAATAAAAAAAAAATATCTGAAAATATAGAGATAGAAAGATAGAGAAAAAAAATAGAATAAGAATATATATCTATCTATAATGTAGTGGATGAAAATATCTAAGAAACAGTCTAGTTTTTTATTTGAAAATTTCTTGTATTGTCAGTTGATCTTTTTTTTTCTTTTTTGCT
