# Supplementary material for: Analysis of Phenolic Compounds of Reynoutria sachalinensis and Reynoutria japonica Growing in the Russian Far East
Source: Plants (Basel). 2024 Nov 27;13(23):3330. doi: 10.3390/plants13233330 (PMC11644227; doi:10.3390/plants13233330)
Supplement: Supplementary file 1 [file plants-13-03330-s001.zip › Figure S3.docx]

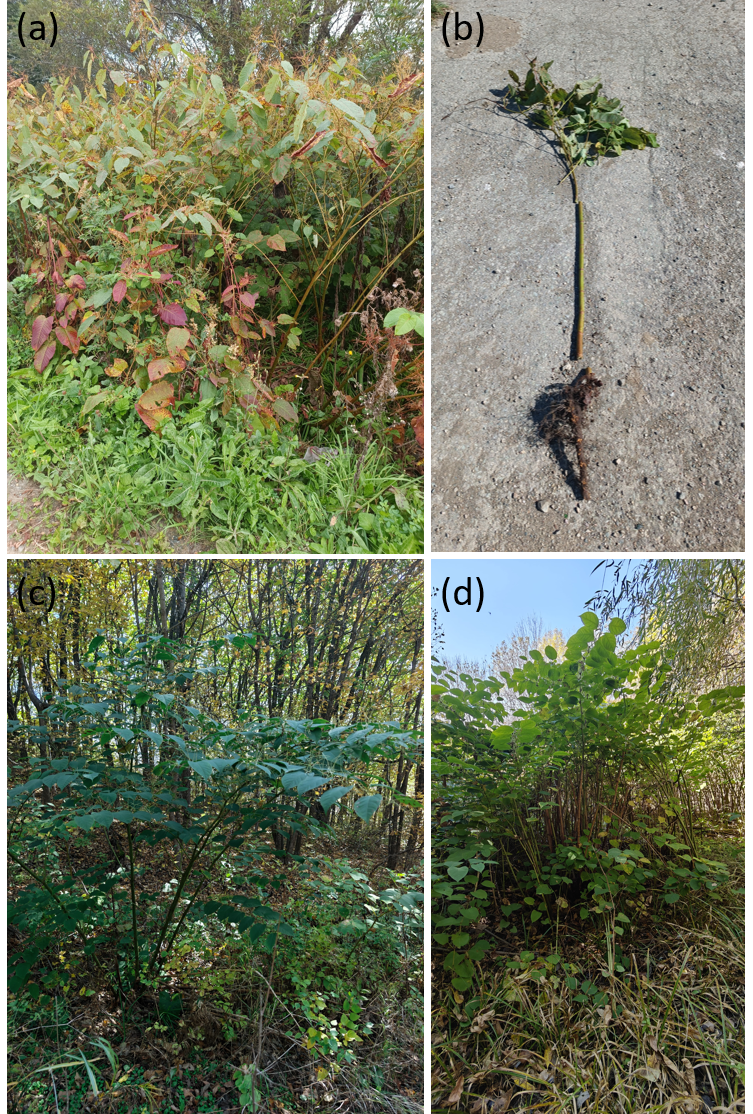


*Figure S3.* Photographs of Reynoutria plants. (**a**) One of the Reynoutria plants collected in Sakhalin Oblast, Russia; (**b**) Photo of analyzed parts of the Reynoutria plant; (**c,d**) Reynoutria plants collected in Primorsky Krai, Russia.
