# Supplementary material for: Analysis of Phenolic Compounds of Reynoutria sachalinensis and Reynoutria japonica Growing in the Russian Far East
Source: Plants (Basel). 2024 Nov 27;13(23):3330. doi: 10.3390/plants13233330 (PMC11644227; doi:10.3390/plants13233330)
Supplement: Supplementary file 1 [file plants-13-03330-s001.zip › Table S1.docx]

Table S1. Reynoutria plant material collected for DNA and secondary metabolite analysis in autumn 2023.

| # | The place of collection of the material | Longitude and latitude |
| --- | --- | --- |
| 1 | Russia, Anivsky urban district, Sakhalin region | 46.771735 and 142.520658 |
| 2 | Russia, Kholmsky urban district, Sakhalin region | 47.057867 and 142.130470 |
| 3 | Russia, Korsakovsky urban district, Sakhalin region | 46.858812 and 143.107846 |
| 4 | Russia, Anivsky urban district, Sakhalin region | 46.854018 and 142.571455 |
| 5 | Russia, Vladivostok city, Akademgorodok, #1 | 43.19061 and 131.92438 |
| 6 | Russia, Vladivostok city, Akademgorodok, #2 | 43.19041 and 131.92421 |
| 7 | Russia, Vladivostok city, Russkay str. #1 | 43.18605 and 131.91501 |
| 8 | Russia, Vladivostok city, Russkay str. #2 | 43.18597 and 131.91469 |
| 9 | Russia, Vladivostok city, Chapaeva str. #1 | 43.16631 and 131.92015 |
| 10 | Russia, Vladivostok city, Chapaeva str. #2 | 43.16634 and 131.92117 |
| 11 | Russia, Vladivostok city, Morgorodok, #1 | 43.14282 and 131.90707 |
| 12 | Russia, Vladivostok city, Morgorodok, #2 | 43.14288 and 131.90701 |
| 13 | Russia, Primorsky Krai, Gornotaezhnoye village, #1 | 43.69727 and 132.15556 |
| 14 | Russia, Primorsky Krai, Gornotaezhnoye village, #2 | 43.69686 and 132.15455 |
