# Supplementary material for: Analysis of Phenolic Compounds of Reynoutria sachalinensis and Reynoutria japonica Growing in the Russian Far East
Source: Plants (Basel). 2024 Nov 27;13(23):3330. doi: 10.3390/plants13233330 (PMC11644227; doi:10.3390/plants13233330)
Supplement: Supplementary file 1 [file plants-13-03330-s001.zip › Table S2.docx]

Table S2. The primer sequences for plant material identification.

| Marker | Genome | Primer, 5ꞌ-3ꞌ | Size (bp) |
| --- | --- | --- | --- |
| ITS | Nuclear | ITS_S, GAGAGCAGAAAGACCCGCG  ITS_A, GATCTGGGGTCGCAACGGT | 550-570 |
| matK | Chloroplast | matK_S, CTACAGTTTACTAGTTGTAAAACG  matK_A, CTCAAACTTCTTAATACCACTATC | 925-931 |
| rps16-trnK | Chloroplast | rps16_trnK_S, GTAGGAGGCTGTGTCTCC  rps16_trnK_A, CGAAATTAGACAAGCCAACC | 708-822 |
