# Supplementary material for: Analysis of Phenolic Compounds of Reynoutria sachalinensis and Reynoutria japonica Growing in the Russian Far East
Source: Plants (Basel). 2024 Nov 27;13(23):3330. doi: 10.3390/plants13233330 (PMC11644227; doi:10.3390/plants13233330)
Supplement: Supplementary file 1 [file plants-13-03330-s001.zip › Table S3.docx]

| **№** | **Rt (min)** | **Negative ion mode** | | **Positive ion mode** | | **UV, λ max (nm)** | **Assignment** | **Group** |
| --- | --- | --- | --- | --- | --- | --- | --- | --- |
|  |  | **[M-H]**  **(m/z)** | **MS2 fragmentation [M-H] (m/z)** | **[M+H]**  **(m/z)** | **MS2 fragmentation [M+H] (m/z)** |  |  |  |
| 1 | 13.0 | 352.7 | 178.6;190.6 | 354.8 | 162.7 | 210, 324 | Chlorogenic acid | Phenolic acids |
| 2 | 14.9 | 336.7 | 162.6; 190.5 | 338.8 | 178.8; 234.6; 277.7; 341.9 | 225; 310 | Coumaroyl quinic acid (isomer of 758, 759,760) | Phenolic acids |
| 3 | 16.0 | 352.8 | 190.6 | 354.8 | 162.7 | 210, 324 | 5-O-Caffeoylquinic acid | Phenolic acids |
| 4 | 17.1 | 577 | 288.7;406.8;424.8 | 579.1 | 290.9;300.8;  409;426.9 | 279 | Procyanidin dimer, Type B | Flavan-3-ols |
| 5 | 18.0 | 288.7 | 244.7 | 290.7 | 150.6; 164.7; 272.7 | 225, 280 | Epicatechin | Flavan-3-ols |
| 6 | 18.7 | 336.8 | 190.6;172.6;  162.6 | 338.8 | 164.7; 322.8; 341.8 | 225; 312 | Coumaroyl quinic acid (isomer of 758, 759,760) | Phenolic acids |
| 7 | 18.9 | 289.6 | 136.5;160.6;202.5;244.5;270.6 | 290.7 | 138.8; 164.7 | 225, 280 | Catechin | Flavan-3-ols |
| 8 | 19.1 | 404.9 | 244.6; 204.5; 178.6 | 406.9 | 244.7; 226.7; 310.8; 226.7 | 305 | Piceatannol 3'-O-glucoside | Stilbenes |
| 9 | 19.8 | 388.8 | 227.7 | 390.9 | 228.7;164.7;  210.7 | 305 | Resveratroloside | Stilbenes |
| 10 | 20.2 | 336.8 | 190.6; 172.6; 162.6 | 338.8 | 162.7; 341.9 | 225; 308 | Coumaroyl quinic acid (isomer of 758, 759,760) | Phenolic acids |
| 11 | 20.5 | 446.8 | 285; 326.6 | 449 | 287 | 347 | Luteolin 7-O-glucoside | Flavones and Flavonols |
| 12 | 21.9 | 388.8 | 226.6;160.6 | 390.9 | 228.7; 210.7; 182.7; 164.6 | 222; 318 | *trans*-Polydatin | Stilbenes |
| 13 | 22.7 | 440.9 | 288.7;244.6 | 443 | 150.7;272.8 | 278 | Epicatechin-3-gallate | Flavan-3-ols |
| 14 | 23.9 | 462.8 | 300.5; 178.5 | 465 | 302.8 | 350 | Quercetin-3-O-glucoside | Flavones and Flavonols |
| 15 | 24.8 | 432.9 | 300.7; 254.4; 178.6 | 434.9 | 302.7 | 257; 352 | Quercetin-3-O-pentoside | Flavones and Flavonols |
| 16 | 25.0 | 446.9 | 300.6; 270.6; 254.4; 178.6 | 448.9 | 302.7 | 257; 349 | Quercitrin | Flavones and Flavonols |
| 17 | 25.8 | 432.8 | 300.6; 178.6; 150.6 | 434.9 | 302.7 | 257; 352 | Quercetin-3-O-pentoside | Flavones and Flavonols |
| 18 | 26.0 | 388.9 | 226.5 | 390.9 | 228.7; 182.7; 164.6 | 222; 285 | *сis*-Polydatin | Stilbenes |
| 19 | 27.0 | 447 | 285 | 449 | 287 | 345 | Kaempferol-3-O- glucoside | Flavones and Flavonols |
| 20 | 30.1 | 226.7 | 226.6; 184.6 | 229 | 211; 135 | 220, 308 | Resveratrol | Stilbenes |
| 21 | 31.1 | 300.6 | 150.5; 178.5 | 303 | 229; 153 | 360 | Quercetin | Flavones and Flavonols |
| 22 | 32.4 | 407 | 244.7 | 409.1 | 246.8 | 226, 325 | Torachrysone glucoside | Naphthalene Derivative |
| 23 | 32.7 | 431.2 | 268.7;226.7 | 433.1 | 271 | 250,270, 426 | Emodin glucoside | Anthraquinones and derivatives |
| 24 | 33.4 | 516.9 | 472.9 | 519.1 | --- | 220, 282, 423 | Emodin-8-O-(6’-O-malonyl)-D-glucoside | Anthraquinones and derivatives |
| 25 | 33.9 | 779.2 | 452.9; 486.9; 633.1 | 601.1 | 178.7; 228.7; 272.7; 418.9 | 221, 312 | Hydropiperoside | Anthraquinones and derivatives |
| 26 | 34.5 | 444.7 | 282.6 | 447 | 285 | 222, 266, 430 | Physcion-8-O-β-D-glucoside | Anthraquinones and derivatives |
| 27 | 34.8 | 244.7 | 227.6; 203 | 246.8 | 229; 205 | 231, 314 | Torachrysone | Naphthalene Derivative |
| 28 | 38.3 | 821.2 | 675, 453 | 845.2 | 601 | 220, 310 | Vanicoside C | Phenylpropanoid Disaccharide Esters |
| 29 | 40.0 | 955.3 | 477, 663 | 979.2 | 601 | 230;314 | Vanicoside B | Phenylpropanoid Disaccharide Esters |
| 30 | 41.0 | 282.9 | 240 | 285 | 270 | 220; 285; 430 | Questin | Anthraquinones and derivatives |
| 31 | 45.1 | 268.7 | 225, 241 | 271 | 229 | 220; 288; 430 | Emodin | Anthraquinones and derivatives |

**Table S3.** List of compounds identified in leaves, stems and roots of *Reynoutria japonica* and *Reynoutria sachalinensis*.
