# Supplementary material for: Analysis of Phenolic Compounds of Reynoutria sachalinensis and Reynoutria japonica Growing in the Russian Far East
Source: Plants (Basel). 2024 Nov 27;13(23):3330. doi: 10.3390/plants13233330 (PMC11644227; doi:10.3390/plants13233330)
Supplement: Supplementary file 1 [file plants-13-03330-s001.zip › Table S4.docx]

|  | *rps16* | *matK* | *ITS2* |
| --- | --- | --- | --- |
| Fallopia japonica | GenBank: JF831293.1 | GenBank: OR464937.1 | GenBank: ON557647.1 |
| Reynoutria elliptica | GenBank: ON586922.1 | GenBank: ON586892.1 | GenBank: ON557643.1 |
| Fallopia convolvulus | GenBank: ON586915.1 | GenBank: ON586882.1 | GenBank: AF040064.1 |
| Fallopia aubertii | Gene ID: 76826202 | GenBank: MH660128.1 | GenBank: HM357909.1 |
| Fallopia baldschuanica | GenBank: ON586913.1 | GenBank: ON586880.1 | GenBank: AF040063.1 |
| Fallopia dentatoalata | Gene ID: 84363420 | GenBank: MN273658.1 | GenBank: EU580726.1 |
| Fallopia multiflora | GenBank: ON586928.1 | GenBank: MH321099.1 | GenBank: ON557648.1 |
| Fallopia sachalinensis | GenBank: JF831294 | GenBank: ON586899 | GenBank: AF040073.1 |
| Fallopia scandens | GenBank: ON586919.1 | GenBank: ON586886.1 | GenBank: ON557637.1 |
| Polygonum aviculare | Gene ID: 68662342 | GenBank: OR463363.1 | GenBank: GQ339983.1 |

Table S4. Sequences of *ITS2*, *matK* and *rps16* genes belonging to the genera Reynoutria and Fallopia were obtained from the NCBI GenBank database
